# Timely delivery of cardiac mmRNAs in microfluidics enhances cardiogenic programming of human pluripotent stem cells

**DOI:** 10.3389/fbioe.2022.871867

**Published:** 2022-08-10

**Authors:** Anna Contato, Onelia Gagliano, Michael Magnussen, Monica Giomo, Nicola Elvassore

**Affiliations:** ^1^ Department of Industrial Engineering, University of Padova, Padova, Italy; ^2^ Veneto Institute of Molecular Medicine, Padova, Italy; ^3^ GOSICH Zayed Centre for Research Into Rare Disease in Children, University College London, London, United Kingdom

**Keywords:** mmRNAs, microfluidics, heart-on-chip, cardiogenesis, hPSCs

## Abstract

In the last two decades lab-on-chip models, specifically heart-on-chip, have been developed as promising technologies for recapitulating physiological environments suitable for studies of drug and environmental effects on either human physiological or patho-physiological conditions. Most human heart-on-chip systems are based on integration and adaptation of terminally differentiated cells within microfluidic context. This process requires prolonged procedures, multiple steps, and is associated with an intrinsic variability of cardiac differentiation. In this view, we developed a method for cardiac differentiation-on-a-chip based on combining the stage-specific regulation of Wnt/β-catenin signaling with the forced expression of transcription factors (TFs) that timely recapitulate hallmarks of the cardiac development. We performed the overall cardiac differentiation from human pluripotent stem cells (hPSCs) to cardiomyocytes (CMs) within a microfluidic environment. Sequential forced expression of cardiac TFs was achieved by a sequential mmRNAs delivery of first *MESP1*, *GATA4* followed by *GATA4*, *NKX2.5*, *MEF2C*, *TBX3*, and *TBX5*. We showed that this optimized protocol led to a robust and reproducible approach to obtain a cost-effective hiPSC-derived heart-on-chip. The results showed higher distribution of cTNT positive CMs along the channel and a higher expression of functional cardiac markers (*TNNT2* and *MYH7*). The combination of stage-specific regulation of Wnt/β-catenin signaling with mmRNAs encoding cardiac transcription factors will be suitable to obtain heart-on-chip model in a cost-effective manner, enabling to perform combinatorial, multiparametric, parallelized and high-throughput experiments on functional cardiomyocytes.

## Introduction

Cardiovascular disease remains a leading cause of morbidity and mortality in global health, with ischemic heart disease representing the majority of deaths over the past 10 years. The high disease burden, both immediate and chronic, imposes great costs onto healthcare systems which necessitate the development of novel therapeutic strategies (Roth et al., 2020). The main issue of current pharmacological and interventional therapeutic approaches is their inability to compensate the great and irreversible loss of functional cardiomyocytes (CMs), due to the very limited supplies of cardiac cells for dedicated studies (Chan and Huang, 2021).

In the recent years organ-on chip and in particular heart-on-chip have been developed to predict response to a multitude of stimuli including drug and environmental effects on human physiological or patho-physiological conditions. These micro-physiological system are mimicking, to some extent, structural and functional characteristics of human cardiac tissue (Luni et al., 2014; Martewicz et al., 2018). Their small scale enables precise control of culture conditions and high-throughput experiments, which would not be economically sustainable on a macroscopic level. The intrinsic properties of microfluidic-based technologies, which involve fluid flow in microscale channels, enable precision spatiotemporal control of fluid dynamics, mass transport and, consequently, the delivery of soluble factors or micro-particles over cell layers. Indeed, they are well suited to control the self-organization and dynamic reciprocity between cell culture of the surrounding microenvironment (Giobbe et al., 2015; Park et al., 2015; Tonon et al., 2019).

Heart-on-chip *in vitro* models mainly rely on the use of primary adult cardiomyocytes. These cells are post mitotic and cannot be expanded *in vitro* for many passages, thus they represent a limited cardiomyocyte source for heart-on-chip applications. Typically, adult cardiomyocytes are integrated at high density within a microfluidic environment (Zhang et al., 2020).

Alternatively, human embryonic stem cell (hESCs) and human induced pluripotent stem cells (hiPSCs) have emerged as effective human cell sources because they can be indefinitely expanded in culture and differentiated into cardiac cells (Burridge et al., 2012). Moreover, deriving hiPSCs from the reprogramming of somatic cells of patient-specific genetic disorders allows researchers to perform a powered study for associating patient-genotype to *in vitro* phenotypes in heart-on-chip model (Pane et al., 2016; Rana et al., 2019).

In this view, research strategies have made several efforts to develop methods to efficiently program human pluripotent stem cells (hPSC) toward cardiac differentiation. The existing methods for deriving CMs involve stage-specific perturbations of different signalling pathways using growth factors (GFs) or small molecules that recapitulate key steps of the cardiac development observed *in vivo* (Lian et al., 2014; Palpant et al., 2017). These strategies are often limited by high intra- and inter-experimental variability. This is mostly caused by the intrinsic variability of starting hiPSC lines, which show differences in the endogenous expression of secreted cytokines or in intrinsic regulation of specific signalling pathways (Volpato and Webber, 2020). For instance, differentiation of CMs from hiPSCs is critically dependent upon the regulation of the Wnt signalling pathway.

An alternative strategy to produce human cardiomyocytes are based on the overexpression of cardiac TFs for the direct lineage conversion of somatic cells. This can be done by integrating and non-integrating vectors (Ieda et al., 2010). However, these approaches are characterized by low efficiencies, combined with further risk of genomic integration and insertional mutagenesis when using integrating vectors, or the need for stringent steps of cell purification and sorting when using non-integrating techniques.

**FIGURE 1:**
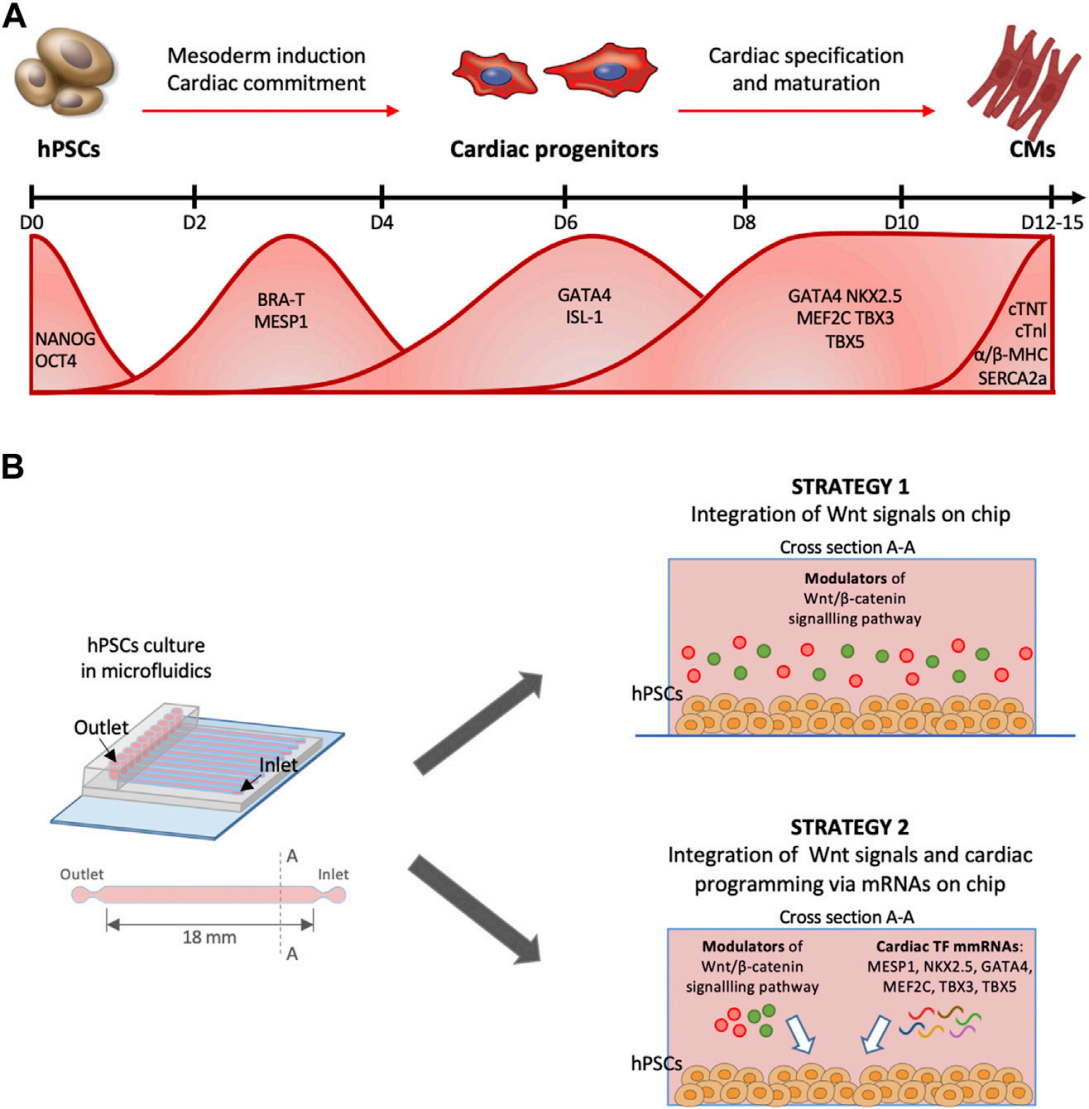
Timing of cardiomyocyte differentiation from hPSCs. **(A)** Timeline of cardiac differentiation steps from pluripotent stem cells. Bottom, waves of expression of the principal cardiac TFs during *in vitro* cardiac differentiation from pluripotent stem cells. **(B)** Schematic protocol for hPSCs cardiac programming implementing two strategies in microfluidics. First strategy provides the integration of Wnt/β-catenin pathway modulation, the second one the combination between Wnt/β-catenin signals and a forced sequential endogenous protein expression *via* cardiac mmRNA delivery.

**FIGURE 2:**
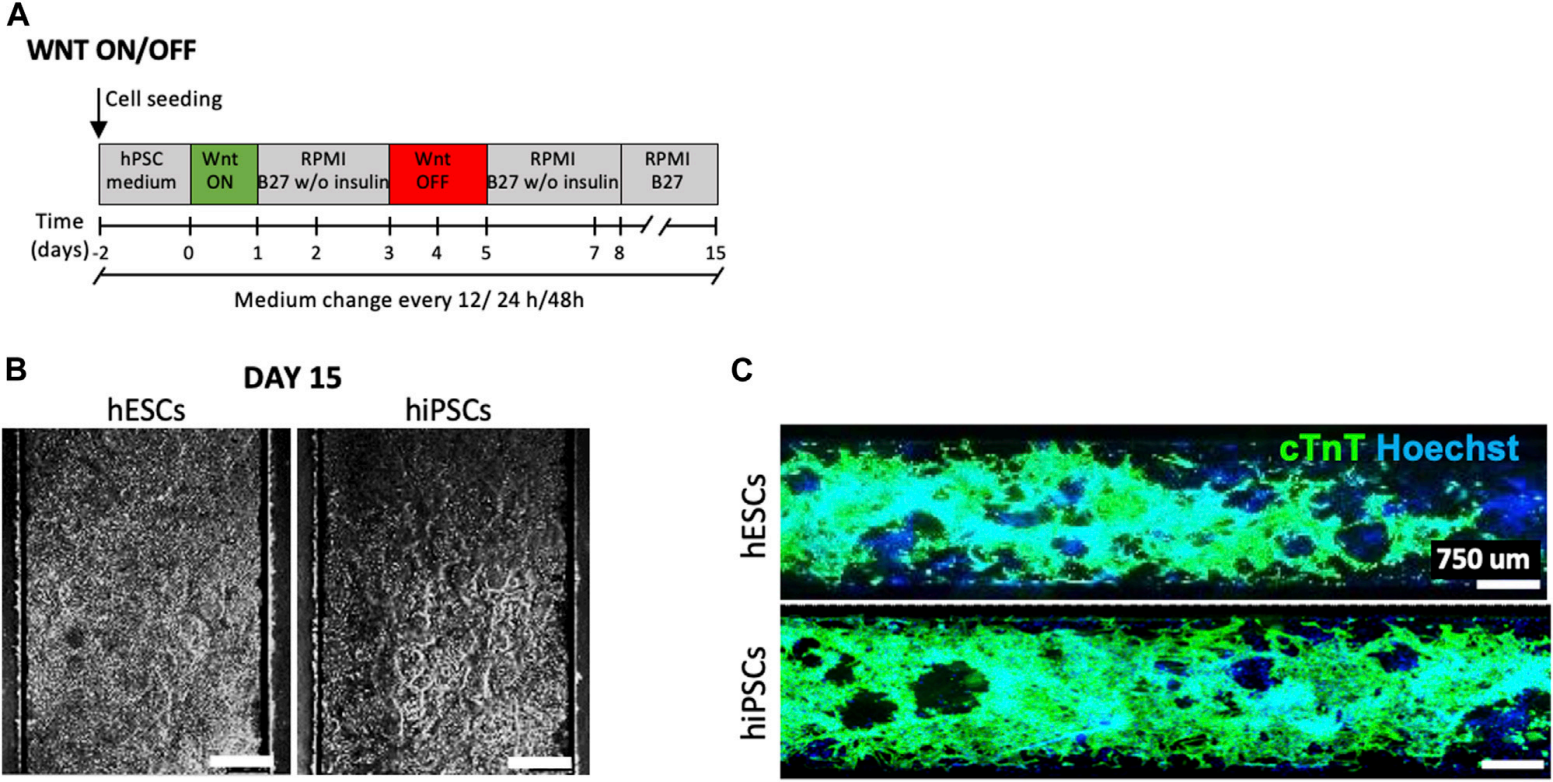
Cardiomyocyte differentiation on chip. **(A)** Schematic representation of the standard protocol based on biphasic role of Wnt/β-catenin signaling (Lian X, 2012) adaptated and optimized into the microfluidic platform. The medium change has been performed every 12, 24, and 48 h. **(B)** Longitudinal section of a microchannel with CMs at day 15 of cardiac differentiation of hESCs and hiPSCs, medium change of 12 h and ECM 2.5%. Scale bars 450 μm. **(C)** Immunofluorescence staining for cTNT (green) in CMs obtained from hESCs (upper panel) and hiPSCs (lower panel) differentiated after optimization of the standard protocol in microfluidics (medium change every 12 h). Nuclei were counterstained with Hoechst. Scale bars 750 μm.

In this work, we aimed at developing a method for cardiac differentiation on a chip based on combining the stage-specific regulation of cardiac signalling pathways with the forced expression of transcription factors (TFs) that recapitulate hallmarks of the cardiac development (Figure 1A). Different models of heart-on-chip have been already proposed from our previous work (Martewicz et al., 2018) and other colleagues (Agarwal et al., 2013; Zhang et al., 2013; Marsano et al., 2016; Zhao et al., 2019), however these studies are based on terminally differentiated cells that are integrated into microfluidic platforms. We performed the overall cardiac differentiation from hPSCs to CMs within a microfluidic environment (Figure 1B). This strategy opens many experimental challenges, such as optimizing culture conditions during differentiation and defining a proper temporal sequence of regulation of signalling pathways and expression of TFs. Moreover, to allow a transient expression of transcription factors restricted to a specific window of time, we decided to use synthetic modified messenger RNAs (mmRNAs). We showed that this microfluidic mmRNA delivery was successful for inducing transcriptional programming of hPSC toward skeletal muscle (Selmin et al., 2021) and neuronal (Tolomeo et al., 2021) programming. Here we were able to transiently express TFs in sequential manner mimicking their temporal profile during cardiac development. This led to a robust and reproducible approach to obtain a cost-effective hiPSC-derived heart-on-chip (Figure 2A).

## Results

### Optimization of cardiac differentiation on-a-chip with Wnt modulators

First, we focused on the adaptation and optimization of the standard protocol based on biphasic roles of Wnt/β-catenin signaling (Lian et al., 2012; Burridge et al., 2014) within the microfluidic platform (Figure 2A). This protocol is designed to recapitulate the hiPSC-CM differentiation during embryonic development and is based on a first phase of Wnt activation (Wnt ON) and a second phase of Wnt inhibition (Wnt OFF). Wnt ON induces the expression of mesendodermal markers, such as Brachyury (*TBXT*) and Eomesodermin (*EOMES*), which directly activate the primary cardiac mesoderm regulator mesoderm posterior bHLH transcription factor 1 (*MESP1*). On the other hand, Wnt OFF mimics the negative regulation of Wnt pathways mediated by the activation of Dickkopf Wnt Signaling Pathway Inhibitor 1 (*DKK1*) by *MESP1*, which drives cardiac-lineage specification.

It is well known that cell behaviour and stem cell fate decisions importantly depend also on extracellular matrix (ECM) interactions that influence early cardiac development (Zhang et al., 2012). Thus, we optimized the coating of microfluidic channels with extracellular matrix (ECM) proteins using 2.5% (vol/vol in DMEM) Matrigel Reduced Growth Factor (MRGF).

Then, we optimized the frequency of medium changes (every 12, 24, and 48 h) in order to define the condition that promotes efficient cell adhesion and allows homogenous cell proliferation and differentiation over all the channel surface (Supplementary Figure S1), using both hESCs (H9) and hiPSCs derived from BJ fibroblasts (Luni et al., 2016). Figure 2B shows a representative example of CMs at day 15 of differentiation in microfluidics, for both hESCs and hiPSCs, obtained with an optimized protocol based on 2.5% v/v MRF and medium replacement every 12 h. Figure 2C shows homogeneous distribution of cardiac troponin T (cTNT) positive cells overall microfluid channel surface.

### Guiding of programming human pluripotent stem cells into cardiomyocytes with mmRNAs encoding cardiac transcription factors in microfluidics

After adaptation of the biphasic regulation of Wnt/β-catenin signaling in microfluidics, we developed a strategy to force the expression of key cardiac TFs for programming cell fate and identity (Burridge et al., 2012; Burridge et al., 2014) toward cardiac lineage. According to TFs that have a relevant role during specification in cardiac mesoderm (Figure 1) during embryonic development (Bruneau, 2013), we selected the following mmRNAs as candidates: *MESP1*, *GATA4*, *NKX2.5*, *MEF2C*, *TBX3,* and *TBX5*. In the first round of experiments, to maintain the experimental complexity at minimum, we decided to deliver all the mmRNAs simultaneously for 6 days (from day 2 to day 8).

**FIGURE 3:**
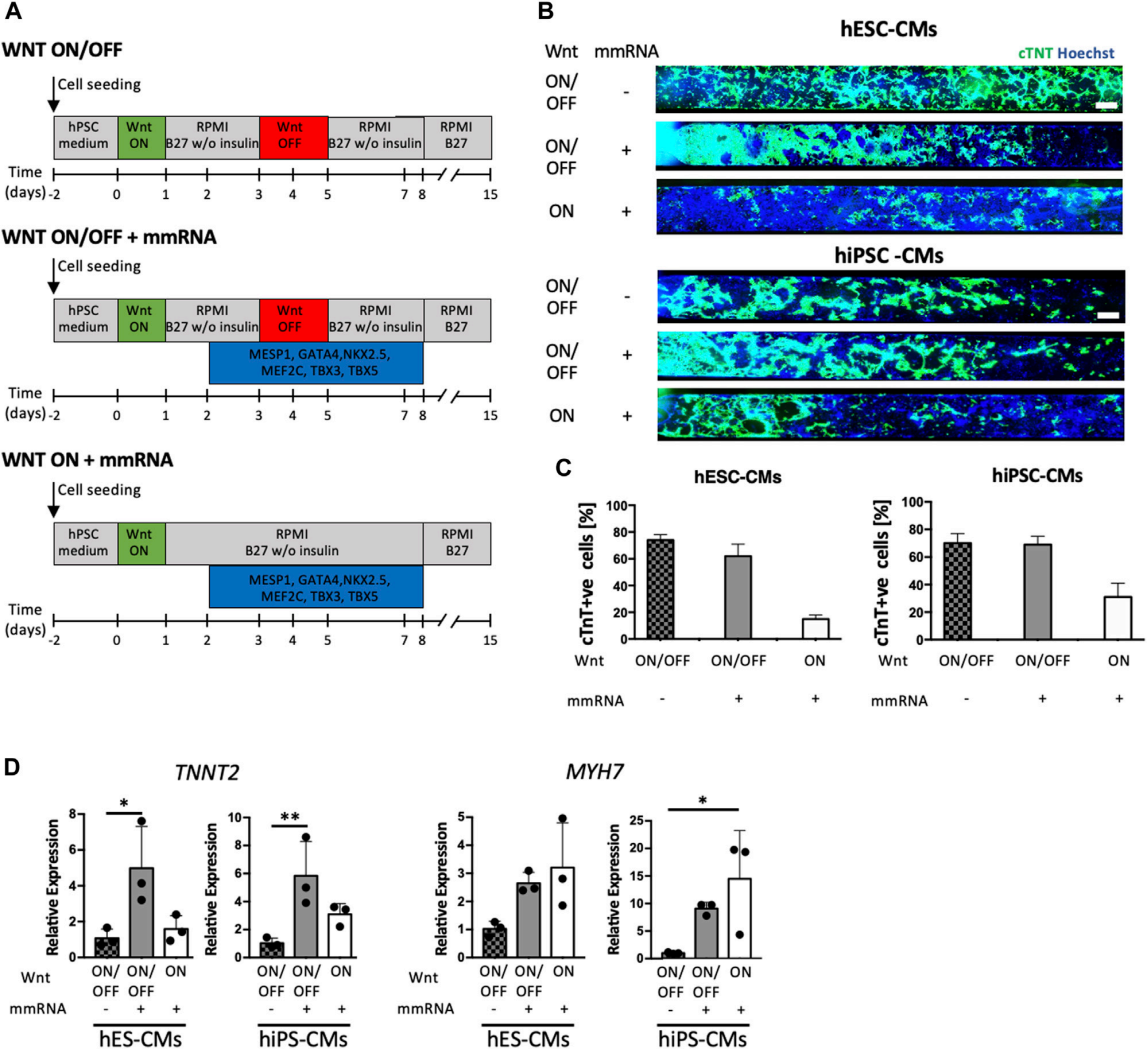
hPSC cardiac differentiation on-chip mediated by Wnt signal and cardiac mRNA expression. **(A)** Schematic representation of the three experimental strategies applied in parallel. Top, standard protocol (Lian X, 2012) for cardiac differentiation with small molecules modulating Wnt/β-catenin pathway, CHIR99021 and IWP4, are used. Middle, strategy WNT ON/OFF + mmRNA in which the complete Wnt/β-catenin pathway perturbation with CHIR99021 and IWP4 was coupled to cardiac mmRNA transfections for 6 days. Bottom, strategy WNT ON + mmRNA which only CHIR99021 was administered, in combination with mmRNA transfections for 6 days. Medium change was performed every 12 h. **(B)** Immunofluorescence staining for cTNT (green) in CMs obtained from hESCs (upper panel) and hiPSCs (lower panel) differentiated in microfluidic platform, in the three protocols implemented. Nuclei were counterstained with Hoechst. Scale bars 750 μm. **(C)** Quantification of cTNT positive CMs from hESCs and iPSCs in the three protocols implemented. **(D)** Gene expression normalized to *GAPDH* of *TNNT2* and *MYH7*, in CMs-mmRNA derived obtained from hESCs and hiPSCs at day 15 of cardiac differentiation. Data are shown as mean, error bars indicate SEM, *n* = 3. One-way ANOVA, *p*-value <0.05 (*); *p*-value <0.01 (**).

Figure 3A illustrates a comprehensive scheme of this experimental setup for simultaneous administration of mmRNAs combined with two protocols: Wnt ON/OFF modulation (using CHIR99021, as Wnt activator, and IWP4, as Wnt inhibitor) and Wnt ON modulation (only using CHIR99021). As control, we used Wnt ON/OFF protocol (Lian et al., 2012; Lian et al., 2013) without mmRNAs delivery. We optimized the lipo-complex based delivery systems and the dose ramp used for providing a highly efficient transfection in microfluidics as reported in Supplementary Figure S2.

In these experiments, we used both H9 and hiPSCs. At day15 cardiomyocytes differentiated in microfluidics were characterized for cardiac markers (cTNT) through immunofluorescence staining (Figure 3B and Supplementary Figure S2B). The cTNT quantification, reported in Figure 3C, revealed that mmRNAs delivery with Wnt ON/OFF protocol led to the same CMs yield of control non-transfected cells. However, interestingly cardiac mmRNAs delivery in Wnt ON protocol was not able to completely replace Wnt inhibition (WNT OFF) that is required for entering in the cardiac differentiation (Figure 3D) program. On the other hand, quantitative RT-PCR showed that mmRNAs led to higher expression of some of the cardiac markers analyzed (*TNNT2* and *MYH7*). Although these results are partially encouraging, they suggested a potential contribution of cardiac TFs in the cardiac differentiation program.

### Timely administration of transcription factor delivery is required for the cardiac differentiation.

Indeed, we asked whether mmRNAs delivery needs to reproduce the timely expression of transcription factors seen during cardiac development (Figure 1). To mimick the temporal pattern of cardiac TF expression, we delivered the TFs in two stages: an “early stage” with *MESP1* and *GATA4* delivered between days 2–4 and a “late stage” with *GATA4*, *NKX2.5*, *MEF2C*, *TBX3,* and *TBX5* delivered between days 4–8.

**FIGURE 4:**
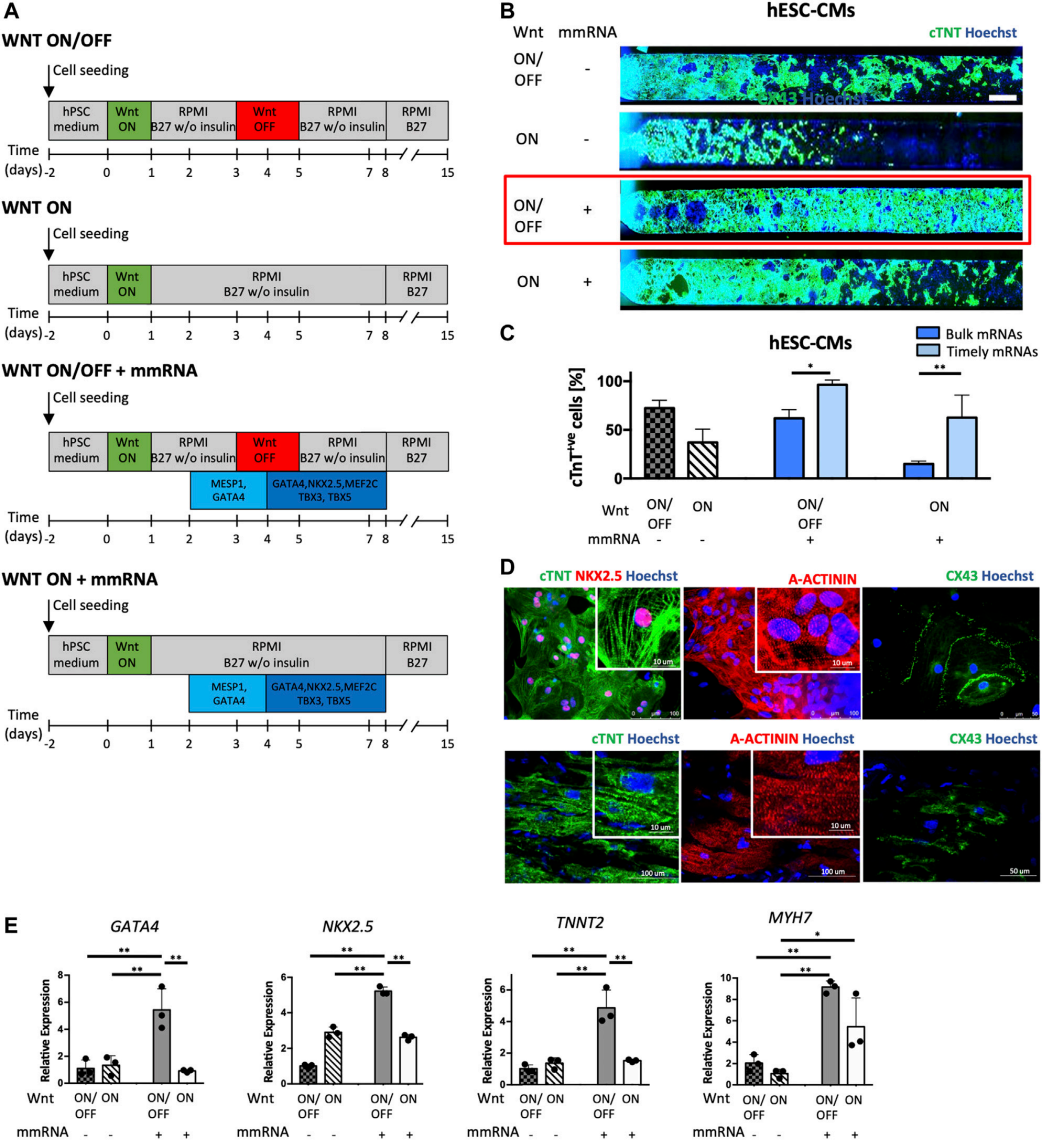
Cardiac differentiation on-chip by combining Wnt modulators and time patterned cardiac mmRNAs delivery. **(A)** Schematic representation of the four experimental strategies applied in parallel. The first two strategies are controls, WNT ON/OFF (Lian X, 2012) and WNT ON respectively, without mmRNA transfections. The other two protocols implement WNT ON/OFF and WNT ON signaling in combination with cardiac mmRNA transfections applied in two phases: early phase (MESP1 and GATA4 for the first 2 days) and late phase (GATA4, NKX2.5, MEF2C, TBX3, and TBX5 for the last 4 days). **(B)** Immunofluorescence staining for cTNT (green) in CMs obtained from hESCs differentiated into microfluidic platform, in the four protocols implemented. Nuclei were counterstained with Hoechst. Scale bar 750 μm. **(C)** Quantification of cTNT positive CMs from hESCs and iPSCs in the four protocols implemented. One-way ANOVA *p*-value <0.05 (*); *p*-value <0.01 (**). **(D)** Confocal images of Immunostaining of cardiac specific proteins acquired; Top, cardiac troponin T (cTNT), NKX2.5, alpha-actinin (A-ACTININ) and connexin 43 (CX-43). Nuclei were counterstained with Hoechst. Bottom, immunostaining of cardiac troponin T (cTNT), alpha-actinin (A-ACTININ) and connexin 43 (CX-43) in human adult cardiomyocytes of the right atrium. **(E)** Gene expression normalized to GAPDH of GATA4, NKX2.5, TNNT2, and MYH7, in CMs-mmRNA obtained from hESCs at day 15 of cardiac differentiation. Data are shown as mean, error bars indicate SEM, *n* = 3. One-way ANOVA *p*-value <0.05 (*); *p*-value <0.01 (**).

Timely delivery of TFs was implemented both in WNT ON/OFF and WNT ON conditions (Figure 4A); the two WNT ON/OFF and WNT ON protocols without mmRNAs were performed as controls.

Notably, from the quantification of cTNT + CMs reported in Figure 4B it is evident that this strategy led a high yield of CMs both in WNT ON/OFF and WNT ON conditions, compared to the controls and the bulk administration of mmRNAs (Figure 4). Combining WNT ON/OFF protocol with timely TFs delivery yielded the highest percentage of cTNT + CMs; interestingly with the modulation of TF delivery it was possible to increase also the number of CMs obtained in WNT ON, indicating a clear effect of mmRNA on protein expression. Moreover, the timely mmRNAs delivery guided in a more effective way the cardiogenesis than the simultaneous administration of TFs (Figure 4C). We characterized the cardiac sarcomere organization in hiPSC-CMs at day 15 obtained in WNT ON/OFF + mmRNAs condition by immunostaining of cTnT, α-actinin, and connexin 43 (Cx43) at the cell surface. CMs at day 15 have been also characterized for the expression of cardiac markers, *GATA4, NKX2.5 (early markers of cardiac mesoderm), TNNT2* and *MYH7 (late cardiac markers)*. Gene expression analysis confirmed that the CMs derived using mmRNAs significantly expressed higher amounts of *TNNT2* and *MYH7* compared with the controls (Figure 4E). These findings, together with the contractile functionality (Supplementary Movie S1), suggested that temporizing the specific mmRNAs delivery in two stages of cardiac differentiation, early and late, resulted in an upregulation of genes (*TNNT2*, *MYH7*) especially involved in CMs structural and functional maturation.

### Characterization of calcium handling and gene expression in mmRNAs-derived cardiomyocytes

To understand the functional maturation of CMs obtained using WNT ON/OFF + mmRNAs protocol we analyzed the Ca^2+^ handling dynamics. This has been associated with the level of maturity of the contractile machinery of differentiated CMs (Martewicz et al., 2017). We investigated the intracellular calcium pattern dynamics using the Fluo-4 as Ca^2+^ probe at day 15 of the differentiation protocol.

**FIGURE 5:**
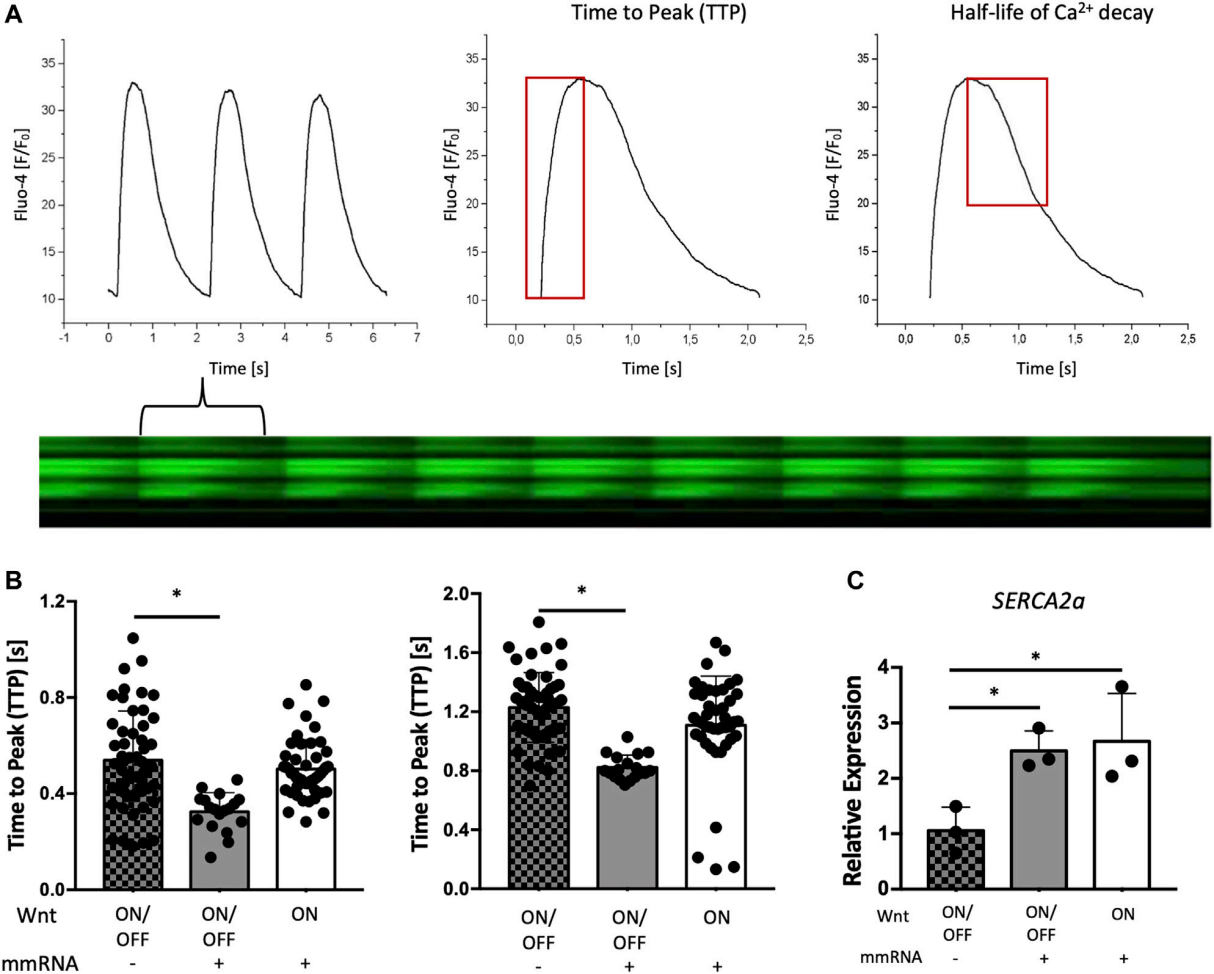
Electrical activity of cardiomyocytes on chip. **(A)** Representative Ca2+ transient profile of in hESCs reporting the fluorescence ratio (F/F0) versus time (s) with the line scan acquisition of the variation of Fluo-4 intensity due to cell contraction and relaxation along time (left panel with the plot at the bottom). From such curves it is possible to evaluate the time to peak (TTP) and half-life of calcium decay. **(B)** Time to peak (TTP) estimates and evaluation of the calcium re-uptake by the half-life of Ca^2+^ decay in hESCs differentiated during the condition of WNT ON/OFF + mmRNA (grey bars), WNT ON + mRNA (white bars), and the control WNT ON/OFF (square pattern grey bars) without mmRNA delivery. Data are presented as mean and error bars represent SEM, *n* = 3. One-way ANOVA *p*-value <0.05 (*). **(C)** Gene expression normalized to *GAPDH* of *SERCA2a* in CMs-mmRNA obtained from hESCs at day 15 of cardiac differentiation, in both WNT ON/OFF and WNT ON conditions with and without delivery of cardiac TFs for 6 days. Data are shown as mean, error bars indicate SEM, *n* = 3. One-way ANOVA *p*-value <0.05 (*).

Figure 5A shows a representative plot of the variation of the fluorescence signal along time, providing the shape of the calcium transient from which it is possible to determine relevant parameters: the time to peak (TTP) value for the calcium release phase and the decay half time to evaluate the calcium re-uptake rate (Figure 5B).

Intriguingly, as shown in the graphs of Figure 5B, calcium transients are significantly faster and shorter (*p* < 0.05) in cells differentiated with WNT ON/OFF + mmRNAs compared with control cells differentiated with the only application of WNT ON/OFF. Also, in CMs obtained with the WNT ON approach and transfected with cardiac mmRNAs the transient is shorter but no significant difference was observed, compared with control cells. These findings clearly demonstrated that the combination of Wnt modulators with the optimized delivery of cardiac mmRNAs, that more closely recapitulate the temporal TFs expression observed during cardiac development, can influence CMs intracellular calcium patterns, indicating an improvement in cardiac functional and maturation features. This increased calcium handling ability, with calcium transient lasting less than 1 s, typical of calcium cycling during contraction, is consistent with maturing CMs features (Lee et al., 2011). Interestingly, *SERCA2A*, a crucial gene for excitation-contraction coupling, was significantly upregulated when the TFs have been administered (Figure 5C). This data is consistent with a faster cytosolic Ca^2+^ clearance by SERCA2a pumps observed in the calcium-induced calcium release analysis. Altogether, these data suggest that CMs generated with WNT ON/OFF protocol with temporized mmRNAs delivery showed high level of expression of structural CM genes involved in cardiac maturation (Figure 4D) with improved functional performance.

## Discussion

In this work, we reported the cardiac programming of hPSC fate with mmRNA technology in microfluidics to generate CMs-on-chip. The association of this novel strategy with a microfluidic platform allowed a precise control and accurate manipulation of mmRNAs and reagents. It was demonstrated that the integration of the experiments into the microfluidic device, thanks to its high surface/volume ratio, determined an increase in the transfection efficiency using 10-fold lower amounts of mmRNAs and reagents compared with standard 24-well plates (Gagliano et al., 2016). Cells were efficiently transfected using two commercial transfection kits based on lipid vehicles, even if one of them showed higher performances.

The use of mmRNAs is a robust and effective tool to force expression of transcription factors and guide cell transcriptomic programming. For deriving greater numbers of terminally differentiated CMs from a limited supply of pluripotent ones, mmRNAs are emerged as a viable option for not only enhancing innate cardiogenic transcriptional programs, but also promoting maturity by forcing programming factors not typically expressed in *in vitro* differentiations. We have previously combined mmRNAs with microfluidics to implement highly efficient cellular reprogramming and programming strategies (Maroof et al., 2013; Giobbe et al., 2015; Luni et al., 2016; Gagliano et al., 2019); although the application of the same strategy of forced endogenous protein expression *via* commercial TFs delivery in a confined environment has never been applied in stem cell cardiac differentiation.

To obtain these results, we developed two strategies for the mmRNAs delivery: the first based on the simultaneous mmRNA delivery and the second one with the timely TFs administration. The six cardiac TFs (*MESP1*, *GATA4*, *NKX2.5*, *MEF2C*, *TBX3*, and *TBX5*) were selected, considering their networking cooperation coming from developmental studies on cardiogenesis *in vivo* (Bruneau, 2013). Furthermore, recapitulating more closely the cardiac developmental stages observed in the embryo, by reflecting the temporal expression windows of each TF has been shown of extreme importance. In fact, it is possible that the forced overexpression of the early-stage cardiac TFs could be detrimental for the late-stage of cardiac differentiation. Indeed, *MESP1*, the master regulator that lies at the hierarchical apex of cardiogenic transcription factor cascades, has been extensively studied for its role in mesodermal patterning. During development, MESP1 is restricted to cells that first egress from the primitive streak (Saga et al., 1999), following which expression takes a notably decline. Strikingly, this expression pattern of MESP1 has been observed to be transient across animal models and *in vitro* systems (Bondue et al., 2008; Chan et al., 2013), and its downregulation is apparently necessary for driving upregulation of cardiogenic transcription factors that pre-empt further differentiation to myocyte subtypes.

Thus, by dividing the transfection regimen in two stages, early and late, it was possible to obtain hiPSC-derived CMs with efficiencies that surpass those obtained in control cells differentiated with only the application of small molecules. This strategy yielded an efficiency of >96% cTNT + CMs when combined with the WNT ON/OFF modulation, whereas simultaneous delivery of all TFs with WNT ON/OFF had a markedly lower efficiency. We attributed this to the continuous exposure of MESP1 from Days 2–8, of which a transient peak in true cardiogenesis at the apex of commitment is required to activate downstream TFs.

Thus, CMs-mmRNAs derived expressed typical cardiac markers and, importantly, they showed significantly faster and shorter calcium transients, indicating an improvement in cell organization, structure, and function toward cell maturation. These results were also confirmed by the analysis of gene expression patterns with qRT-PCR, which showed significantly higher amounts of transcripts than cells differentiated with only Wnt modulators.

Taken together, these experiments provide a proof-of-principle of the possibility to program hPSCs fate toward cardiac lineage by combining the novel technology of synthetic mmRNAs with microfluidics for precise delivery of specific TFs to obtain in a cost-effective manner. Significantly, the technique has powerful potential to robustly compensate the inability of typical hiPSC differentiation strategies inducing all TFs actions without the necessary promotional extracellular factors. We envision combining this method with shortlisted transcriptomic discoveries of single-cell sequencing datasets that identify discrepancies and TF absentees in differentiations, such as the hypertrophic-inducing TF HOPX (Friedman et al., 2018) (non-DNA binding homeodomain protein), a key regulator in cardiac development and hypertrophy. This can make achievable fully programmed cardiomyocytes more representative of the native counterparts that they seek to emulate and supplant.

## Conclusion

The findings developed during this project provide a proof-of-principle that it is possible to program hPSCs fate toward cardiac lineage and cardiac maturation in a seamless process in microfluidics. This hiPSC-based heart-on-chip developed in a cost-effective manner enables performance of multiparametric and parallelized functional assays on patient-specific cardiomyocytes. In addition, the CMs obtained in a confined microfluidic system can be considered clinically compliant grade and could be potentially employed in the next future for clinical applications.

## Materials and methods

### Microfluidic device fabrication and functionalization

Microfluidic devices used in this work were fabricated by standard soft-lithography technique according to the procedure already described in our previous works (Luni et al., 2016; Gagliano et al., 2019). Each microfluidic device contains ten independent channels 18 mm long, 1.5 mm wide and 200 μm high.

An elastomeric stamp made in polydimethylsiloxane (PDMS) is prepared through replica molding by casting a liquid prepolymer and a curing agent solution (Sylgard 184 from Dow Corning) onto a pre-made master with a patterned relief structure built in SU8-2100 negative photoresist (MicroChem) on its surface.

Once cured at 70°C for 2 h, the PDMS mold was cut, peeled off and punched with a 21G stainless steel needle (Small Part Inc.) to obtain inlet and outlet holes. The PDMS mold was assembled and sealed to a 50 mm × 75 mm cleaned glass slide by plasma bonding. Ten independent medium reservoirs (each with a 70 μl capacity), one for each channel, were obtained by sealing an additional PDMS block to the top of the device by plasma bonding. The assembled device was cleaned with isopropanol (Sigma-Aldrich) and sterilized by autoclave, to prevent bacterial contaminations. Glass surface has to be opportunely functionalized with Matrigel Reduced Growth Factor (MRGF; BD Biosciences) in order to promote cell adhesion, survival and growth.

### Cell culture and integration in microfluidic devices

For the experiments two cell lines have been used H9 and hiPSCs derived from BJ fibroblasts. Cell culture in microfluidics is similar to traditional multiwells, concerning the type of medium and reagents used; the unique variables is the volume handling. In the microfluidic platform used in this work, the effective channel volume is 5.4 μl. For the media change, a total volume of 12 μl was perfused to guarantee an extensive medium refreshment and waste products removal.

Prior to cell seeding, microfluidic channels within the chip were filled with 4°C cold Matrigel Reduced Factor® (MRF, BD) 0.5% v/v in DMEM, incubated at room temperature for at least 1 h and washed with StemMACSTM iPS-Brew XF medium (Miltenyi Biotech).

hPSCs were then dissociated in single cell using Tryple (Thermo Fisher Scientific) and injected at 700 cells/mm2 density into the channels in medium supplemented with 10 μM ROCK inhibitor (Y-27632, Miltenyi Biotech) to preserve cell viability.

Because of the reduced medium volume in the microchannels, to prevent a significant evaporation, the microfluidic platform was placed in a 100 mm Petri dish and maintained in an isotonic bath with PBS for an optimal humidity of the chamber.

### Cardiac differentiation on chip

Cardiac differentiation protocol was initiated 2 days after integrating hPSCs into the microfluidic device. Cardiac differentiation was performed according to protocols described by Lian et al. (2012) based on temporal modulation of canonical Wnt/β-catenin signalling pathway.

After 2 days of cell recovery, we implemented the cardiac differentiation using Wnt/β-catenin pathway modulators by using both “WNT ON/OFF” and “WNT ON” protocols.

Differentiation in “WNT ON/OFF” protocol is initiated by removing the IPSBREW medium and replacing it with RPMI 1640 medium (Thermo Fisher Cat.)/B-27 minus insulin (Thermo Fisher) supplied with the GSK3 inhibitor [12 μMCHIR99021 (ABCR, cat. no. AB253776)] for 24 h. Medium was replaced with RPMI 1640/B27-minus insulin for the following 2 days. At day 3 inhibition of canonical Wnt signaling [5 μM IWP4 (Stemgent, cat. No. 04-0036)] in RPMI 1640/B27-minus insulin, is performed for 48 h. From day 5-to day 7 medium was replaced with RPMI 1640/B27-minus insulin, from day 8-to day 15 with RPMI 1640/B27.

In “WNT ON” protocol only 24 h of culture in Basal Medium supplied with the GSK3 inhibitor was provided after removing IPSBREW medium; from day 5-to day 7 medium was replaced with RPMI 1640/B27-minus insulin, from day 8-to day 15 with RPMI 1640/B27.

The mmRNAs (MESP1, GATA4, NKX2.5, MEF2C, TBX3, TBX5) have been delivered within the microfluidic channel for 6 days, from day 2 to day 8, using two kits of transfection, StemMACS (Miltenyi Biotech) and Stemfect (Stemgent) according to the manufacturer’s instructions. All the mmRNAs were produced and provided by Miltenyi Biotec (Cologne, Germany). mmRNAs used are in vitro-transcribed mRNAs that have been carefully optimized to ensure high level expression after transfection. Modified mRNA reduces the innate antiviral response to single-stranded mRNA and robust expression of the encoded factor after transfection has been verified by immunofluorescence or flow cytometry. mmRNAs mix was prepared according to the equimolar ratios of MESP1, GATA4, NKX2.5, MEF2C, TBX3, TBX5 of 1(11.1 ng):2(22.2 ng):1.5(16.7 ng):1.5(16.7 ng):2(22.2 ng):1(11.1 ng) respectively to have 100 ng of total amount of mmRNA. They have been transfected into cells using lipo-complex structures diluted in the media described in WNT ON/OFF and WNT ON protocols.

During transfections the medium was supplemented with B18R, 4 h prior to transfections and added fresh every day. To avoid stressful events for the cells, a transfection dose ramping of medium for the dilution of the transfection mix was always performed, as reported in the Supplementary Figure S2A.

### Immunofluorescence

Immunofluorescence analyses were performed on 4% paraformaldehyde-fixed cells for 15 min. Blocking and permeabilization were performed in blocking solution [5% heat-inactivated FBS and 0.1% TritonX-100 (Sigma-Aldrich) in PBS 1×] for 1 h. The cells were stained then overnight at 4°C using primary antibody of cTNT 1:200 (ThermoFisher Scientific, cat. no. MS295p), NKX2.5 1:100 (Santa Cruz, cat. no. sc-12514) alpha-Actinin 1:1000 (Sigma, cat. no. A7811) and CX-43 1:500 (Sigma, cat. no. C6219) in blocking buffer. Secondary antibodies were diluted in blocking solution and incubated for 2 h. Nuclei were counterstained with HOECHST 33342 (ThermoFisher Scientific, United States). Pictures were taken on Leica DMI6000 B and Leica SP5 microscopes. Efficiency of generating cTNT positive cardiomyocytes was quantified in 10 randomly picked 10x microscopic fields (*n* = 3) after immunostaining with cTNT marker. The efficiency of differentiation was calculated as relative cTNT^+^ cell area divided by the total area occupied by the cells.

### Calcium imaging

Briefly, confocal calcium measurements were performed by loading CMs in serum-free 25 mM HEPES D-MEM (Life Technologies), supplemented with 2.5 μM fluorescent calcium binding dye Fluo-4 AM (Life Technologies). To increase its liposolubility and facilitate the crossing of cell membrane, Fluo-4 was loaded as an acetyl-methyl ester, in presence of 2 μM Pluronic F-127 (Invitrogen) as a mild detergent, for 20 min at 37°C. Cells were then incubated for additional 10 min at 37°C without Fluo-4 for a complete de-esterification of the dye. 20 μM of the anionic transporter inhibitor sulphinpyrazone (Sigma Aldrich) was added in all the media used to limit the dye active extrusion from the cells. As red cell membrane counterstain, 0.2 μM di-8-ANEPPS (Invitrogen) was used in order to distinguish separated cells. Calcium dynamics were acquired in recording solution containing: NaCl 125 mM; KCl 5 mM; Na3PO4 1 mM; MgSO4 1 mM; HEPES 20 mM; CaCl2 2 mM; glucose 5.5 mM, adjusted to pH 7,4 with NaOH (all from Sigma Aldrich). Line scans were acquired with a Leica TCS SP5 confocal microscope equipped with a 63X, 1,4 NA oil immersion objective, with 488 nm Ar laser line as an excitation source, 400 Hz frequency. To avoid or reduce dye photo-bleaching and phototoxic effects on CMs, the laser power was set at minimum possible. All the experiments were conducted at room temperature within 20–30 min from the end of the loading procedures described previously. All data handling and computation was performed with Origin 8.1 software.

### Gene expression analysis by real-time PCR

For gene expression analysis, total RNA from 15 days cells in the microfluidic platform was extracted using iScript (BioRAD), a sample preparation buffer designed to efficiently degrade the cytoplasmic membrane while leaving intact the nuclear membrane; thus only the cytoplasmic RNA was extracted, whereas the genomic DNA remains confined within the nuclei, allowing a sensitive quantitative gene expression analysis. This novel reagent accelerates and streamlines quantitative real-time PCR (qRT-PCR) by eliminating the need for lengthy RNA purification. Briefly, microfluidic channels were perfused with D- PBS prior to 10 μl of iScript injection and the solution was then collected after 1 min of incubation at room temperature. RNA (0.1 μg) was reverse transcribed into cDNA by Reverse Transcription (RT, Life Technologies), according to manufacturer’s instructions.

The qRT-PCR was performed with TaqMan gene expression assay probes (Life Technologies) according to the manufacturer’s instructions. The following genes were used: *GAPDH* (Hs02758991_g1), *GATA4* (Hs01034629_m1), *MYH7* (Hs01110632_m1), *NKX2.5* (Hs00231763_m1) *SERCA2A* (Hs00544877_m1) and *TNNT2* (Hs00165960_m1). Reaction was done on ABI Prism 7000 SDS, Sequence-Detection-System (Applied Biosystems) machine and results were analyzed with ABI Prism 7000 SDS Software. *GAPDH* was used as reference genes to normalize Ct values for gene expression. Data were shown as relative expression to control cells using Livak method (Livak and Schmittgen, 2001).

### Statistical analysis

For each experimental condition at least three independent biological experiments were performed. Statistical significance was assessed by One-way ANOVA-test and presented as the mean ± SEM, determined from at least three independent experiments. For all analysis: *p < 0.05; **p < 0.01; ***p < 0.001; ****p < 0.0001. GraphPad Prism Mac (v. 7.0a) was used to do graphs and statistical analysis.

## Data Availability

The raw data supporting the conclusion of this article will be made available by the authors, without undue reservation.
