# Supplementary material for: Timely delivery of cardiac mmRNAs in microfluidics enhances cardiogenic programming of human pluripotent stem cells
Source: Front Bioeng Biotechnol. 2022 Aug 10;10:871867. doi: 10.3389/fbioe.2022.871867 (PMC9399928; doi:10.3389/fbioe.2022.871867)
Supplement: Supplementary file 2 [file DataSheet1.PDF]

Figure S1

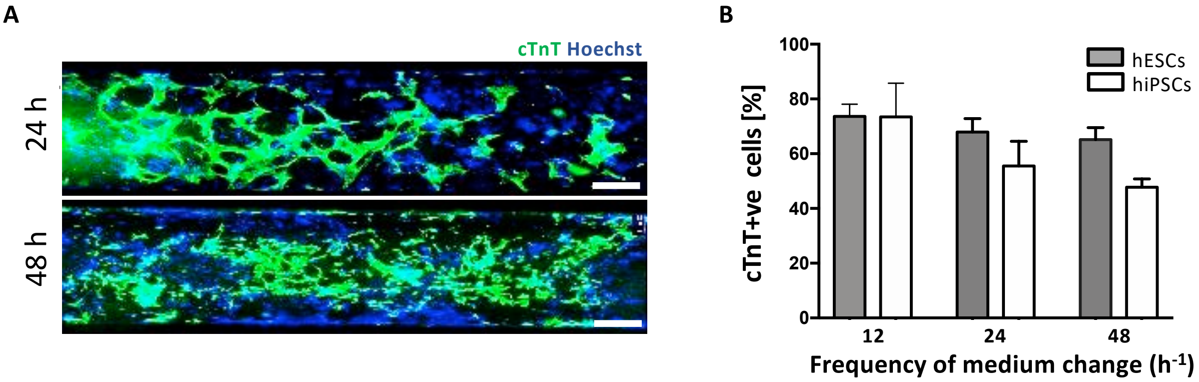

Figure S1: Optimization of cardiac differentiation on-a-chip based on Wnt modulators. (a) Immunofluorescence staining against cardiac markers cTnT in hESC-CMs conditions of 24 h and 48 h of medium change. Nuclei were counterstained with Hoechst. Scale bar 750 µm. (b) Percentage of cTnT+ve CMs derived from hESCs and hiPSCs obtained with in conditions of 12h, 24h and 48h medium change; data are shown as mean, error bars indicate SEM, n=3.

Figure S2

A

Transfection dose-ramp	
**Transfection days**	**Medium/Cardio Mix [%]**
Day 1	Middle dose (50/50%)
Day 2	High dose (25/75%)
Day 3	
Day 4	
Day 5	
Day 6	Middle dose (50/50%)

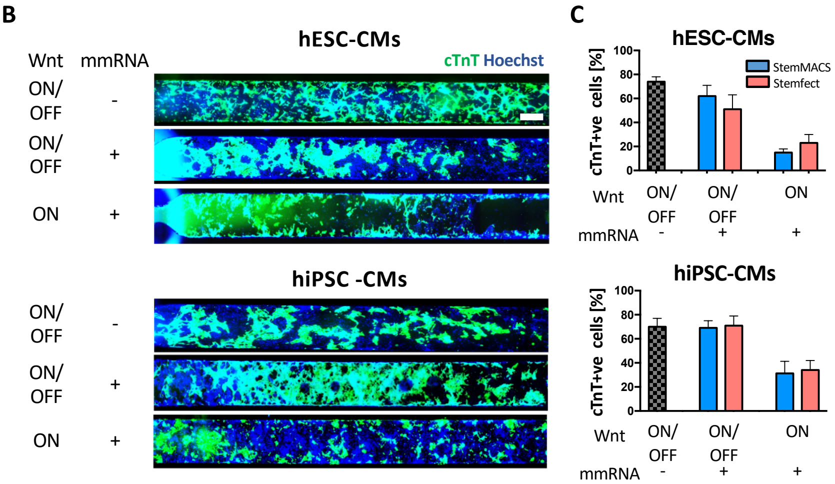

Figure S2: Optimization of cardiac mRNA delivery in microfluidics. (a) Dose ramp used for the cardiac mmRNAs transfection. (b) Immunofluorescence staining for cTnT (green) in CMs obtained from hESCs (upper panel) and hiPSCs (lower panel) differentiated into microfluidic platform, in the three experimental conditions using Stemfect reagent for transfection. Nuclei were counterstained with Hoechst. Scale bar 750 µm. (c) Quantification of cTnT positive CMs from hESCs and iPSCs obtained with Wnt/β-catenin pathway modulators and cardiac mmRNA delivered comparing two different transfection kits, StemMacs (blue bars) and Stemfect (red bars) with the control CMs obtained with Wnt modulators alone (square pattern grey bars). Data are shown as mean, error bars indicate SEM, n=3.
